# Expert Views on Regulatory Preparedness for Managing the Risks of Nanotechnologies 

**DOI:** 10.1371/journal.pone.0080250

**Published:** 2013-11-11

**Authors:** Christian E. H. Beaudrie, Terre Satterfield, Milind Kandlikar, Barbara H. Harthorn

**Affiliations:** 1 Institute for Resources, Environment and Sustainability, University of British Columbia, Vancouver, British Columbia, Canada; 2 Liu Institute for Global Issues, University of British Columbia, Vancouver, British Columbia, Canada; 3 National Science Foundation Center for Nanotechnology in Society, University of California, Santa Barbara, Santa Barbara, California, United States of America; University of Kansas, United States of America

## Abstract

The potential and promise of nanotechnologies depends in large part on the ability for regulatory systems to assess and manage their benefits and risks. However, considerable uncertainty persists regarding the health and environmental implications of nanomaterials, hence the capacity for existing regulations to meet this challenge has been widely questioned. Here we draw from a survey (N=254) of US-based nano-scientists and engineers, environmental health and safety scientists, and regulatory scientists and decision-makers, to ask whether nano experts regard regulatory agencies as prepared for managing nanomaterial risks. We find that all three expert groups view regulatory agencies as unprepared. The effect is strongest for regulators themselves, and less so for scientists conducting basic, applied, or health and safety work on nanomaterials. Those who see nanotechnology risks as novel, uncertain, and difficult to assess are particularly likely to see agencies as unprepared. Trust in regulatory agencies, views of stakeholder responsibility regarding the management of risks, and socio-political values were also found to be small but significant drivers of perceived agency preparedness. These results underscore the need for new tools and methods to enable the assessment of nanomaterial risks, and to renew confidence in regulatory agencies’ ability to oversee their growing use and application in society.

## Introduction

The degree to which scientists, engineers and regulators are prepared for managing the risks of nanomaterials and their derived products is an open question as empirical knowledge of nanoscale materials and their behaviours is emerging at a slow pace [1]. Significant scientific uncertainties related to both the toxicity and exposure characteristics of these materials remain [2]. In the interim, strategies for the regulation of nanomaterials are necessarily emerging through reference to expert, policy and legal advice. How well this is working is, however, largely unknown. This paper thus examines the perceived state of regulatory and agency preparedness from the point of view of key experts.

One early expert study indicated that, overall, nano-experts are more worried about the risks of engineered nanomaterials than are lay or public groups [1,3]. Other work suggests an optimism bias amongst those who develop nano materials and products as compared to those who study or manage their risks [4,5]. Finally, surveys of industry leaders indicate high levels of perceived uncertainty and risk [6], however much they report not following risk-avoidant health and safety practices [7] . 

Studies of expert opinion in earlier and often more controversial risk domains include expert evaluations of chemical risks [8-11], climate change detection and impacts [12-14],expert views on genetically modified organisms (GMOs) [15,16], and expert perception of ecological risks [17,18]. Differences of opinion have been found to vary according to disciplinary fields [10,16] and/or institutional affiliation (e.g., toxicologists in industry versus academia) [9,19]. Expert opinion has also been found to vary significantly with political attitudes and values. Several scholars have found that similar to non-experts, scientists often use norms or values when making judgments about risk under high uncertainty [10,20-22]. For instance, economically conservative nanoscientists were found by Corley et al. to show less support for regulation [22]. Similarly, trust (*in scientists and/or government*) has been found to correlate closely with risk perceptions, with attenuation in perceived risk accompanying higher levels of trust [23,24]. Prominent examples of this effect have also been demonstrated in studies of perceived risk of chemicals and GMOs [15,25]. The effect of attributed stakeholder responsibility, that is, the degree of responsibility assigned to different stakeholders to mitigate or manage risk, has received relatively less attention in the nanotechnology domain. Yet a growing body of literature in public health fields suggests a link between attributions of responsibility and support for government and regulatory policy [26-29]. 

More specifically, nanotechnology researchers have demonstrated differences in perceived *need* [30] and *support* [22] for the oversight of nanotechnologies, which has been explained in reference to experts’ disciplinary degree (i.e. chemistry, physics, materials science, engineering, biology, or other) within the NSE (nano science and engineering) professional body only. Disparity of opinion on risk was also attributed to an observed optimism bias of NSE researchers, compared to NEHS (nano environment, health, and safety) scientists (e.g. toxicologists) [31]. Powell [32] also found significant differences in opinion regarding the novelty and risks of nanomaterials between ‘upstream’ and ‘downstream’ researchers; that is, experts involved in the creation of nanotechnologies, versus those engaged in evaluating the health and environmental implications of ENMs. Yet, nanomaterial novelty remains a relatively untested *driver* of expert perceptions and/or their views regarding risk and regulation. 

No studies have been conducted, however, which classify experts as to their specific role in (1) developing materials versus (2) studying their toxicological behaviour versus (3) assessing and managing their risks. As well, no studies have examined these expert positions as predictive of whether the regulatory system or regulatory agencies are prepared for the oversight of engineered nanomaterials across different technologies, applications or contexts. 

This study thus draws from a systematic sampling of US-based nano-scientists and engineers (NSE, n=114), nano-environmental health and safety scientists (NEHS, n=86), and regulatory decision makers and scientists (NREG, n=54), to characterize how well-prepared different experts think regulatory agencies are for the risk management of nanomaterials and applications. We tested the following hypothesis:

(1) Expert views on whether US federal agencies are sufficiently prepared for managing any risks posed by nanotechnologies will differ significantly across classes of experts (NSE vs. NEHS. vs. NREG).

This difference across experts was anticipated and so tested in reference to four additional hypotheses:

(2) Experts who see nanotechnologies as *novel* (i.e., as a new class of materials or objects) will view US federal regulatory agencies as unprepared for managing risks as compared to those who see nanotechnologies as *not* new (i.e., as little different from their bulk chemical form)
(3) Experts who deem US federal regulatory agencies as less trustworthy will also view agencies as less prepared compared to those with more trust in agencies
(4) Experts who attribute greater collective stakeholder responsibility (e.g. who view a range of stakeholders as equally responsible for managing risks) will see agencies as less prepared compared to those who attribute less responsibility.
(5) Experts who are more socially and economically conservative will see regulatory agencies as more prepared compared to those with a more liberal orientation.

To ensure that measured differences in perceptions of preparedness were not the result of unmeasured differences in demographics and domain of expertise [33], gender, highest degree achieved, year awarded, disciplinary field, and institutional affiliation are included as control variables in this analysis.

## Methods

This research was conducted under the approval of the Behavioural Research Ethics Board, University of British Columbia, and the Institutional Review Board, University of California Santa Barbara. Written informed consent was obtained from all survey respondents. The data reported here were collected through a web-based survey (N=404), designed to assess US & Canadian nanotechnology experts’ perceptions of risks and regulation. The survey was conducted by the University of California Santa Barbara Social Science Survey Center for the UCSB Center for Nanotechnology in Society between June 2nd and November 8th, 2010. To construct the sample frame, we compiled names and detailed contact information for 2,100 experts within three pools of US and Canadian experts: nano scientists and engineers (NSE), nano EHS scientists and toxicologists (NEHS), and scientists and regulators in government agencies (NREG). Subjects were contacted by email in a three-step process, including initial contact and two reminders at two-week intervals. Respondents received an ‘A’ or ‘B’ version of the survey at random, where the wording of several survey questions were modified to reverse the meaning of the question. Questions with alternate wording were reversed-coded during analysis to enable direct comparison of responses. Where appropriate the sequence of questions was also varied to minimize order effects.

For the NSE group, experts were selected using a rigorous sampling design, based on a bibliometric analysis methodology developed by Porter et al. [34] using nanotechnology publications identified through ISI Web Of Science. We excluded papers with the following terms to remove publications that would fall under our NEHS sampling strategy: toxic* or genotoxic* or ecotoxic* or (oxidative stress) or safety or pollution or (environmental health) or (human health) or (animal health) or (public health) or (occupational health). Results were limited to articles and review papers by authors in the US and Canada. 1,200 subjects were selected at random from a pool of over 5,700 first or corresponding authors who published five or more nanotechnology articles that were cited five or more times between 2000 and 2009 (a method utilized by Scheufele et al. (2007)), with at least one article newer than 2006. Database searches were conducted between August and September 2009. 

NEHS experts were selected from first or corresponding authors of 1,600 articles entered into the International Council on Nanotechnology (ICON) Environment, Health and Safety Database between early 2007 and spring 2010. Due to the relatively small domain of nano EHS research, we could not apply the same rigorous NSE standard of selecting authors with five or more publications, and instead selected 500 experts at random from list of over 1,600 authors. International contacts were removed from the list, and several authors listed with .gov email suffixes were cross-referenced with the NREG group for duplications, and removed from the NEHS group.

NREG experts were identified from nanotechnology conference attendance lists, referrals, and website searches of employees in nanotechnology groups in US and Canadian Federal Regulatory Agencies (including EPA, OSHA, FDA, CPSC, Health Canada, Environment Canada) and within Federal research institutes (NIOSH, NIH, national labs), as well as US State regulatory agencies (including Massachusetts Department of Environmental Protection, New York Department of Environmental Conservation, California EPA, North Carolina Department of Environmental and Natural Resources, and Washington Department of Ecology). Contact information and agency affiliation were compiled for 400 NREG experts in spring 2010. A full list of agencies is available in Table S1 in the Supporting Information. 

A total of 404 responses were analyzed, for an overall response rate of 23% (AAPOR RR-3: 23%). Individual group response rates were: NSE: N=180, RR=16%; NEHS: N=121, RR=33%; NREG: N=103, RR=32%. In total 254 participants specified their residence in the US, while 55 reside in Canada, and 95 did not disclose their country of residence, and so might belong to either country. Only the US responses were included in this analysis, Canadian results will be reported in a future publication. For the data reported in this paper, the US sample sizes were: NSE = 114, NEHS = 86, and NREG = 54. We believe the relatively low response rate of the NSE group is due to a large number of outdated mail and email addresses (our search criteria includes publications since 2000). Contacts may have moved institutions or changed email addresses since the date of publication, and therefore were not measured as ‘bounced’ or ‘out-of-scope’. Separate response rates for the US and Canadian groups were not possible since not all respondents indicated their country of residence in their survey responses. 

Statistics were calculated using the SPSS software package [35]. All Principal Component Analyses (PCA) were conducted using orthogonal rotation (Varimax), and scree tests where used to select components [36]. Index scores were calculated using the Anderson-Rubin method unless otherwise specified, producing scores with an overall mean of zero and standard deviation of 1. 

## Results

### Agency Preparedness and Regulator Concern

**Figure 1:**
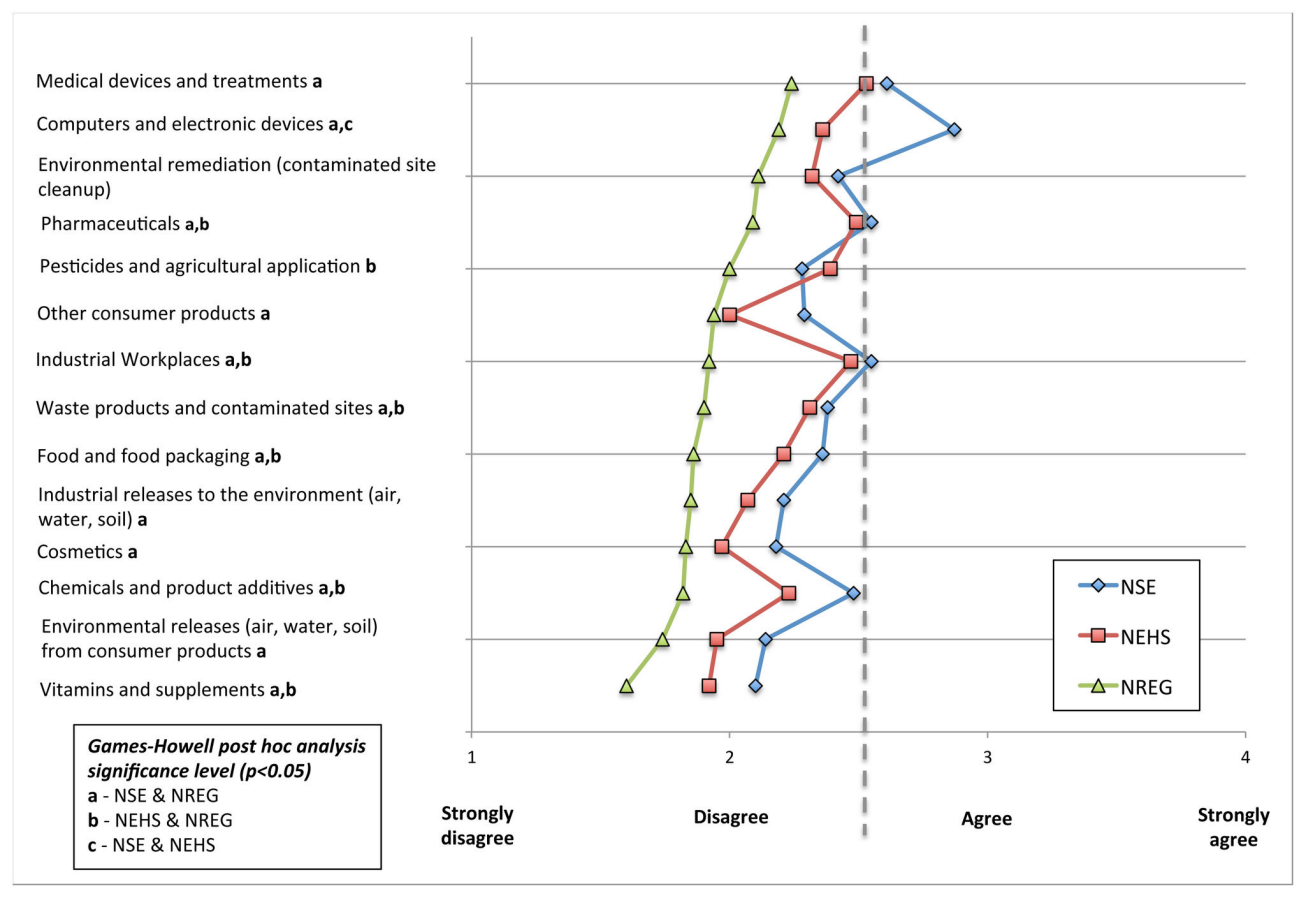
'Agency Preparedness' ratings for NSE, NEHS, and NREG expert groups. Mean scores for each group are indicated with points on respective color-coded lines capturing 14 different nanotechnology scenarios. The dotted grey line indicates the mid or neutral-point between ‘strongly disagree’ and ‘strongly agree’. Significant differences in means were determined using a one-way ANOVA with Games-Howell post hoc analysis, and are indicated with a, b, and c markings as outlined in the legend.

Our expectation that experts would differ significantly as to how prepared regulators are for controlling the risks of nanotechnologies was tested by presenting each respondent with 14 brief descriptions of nanotechnology scenarios. They were asked to rate each using the following scale: “Please indicate whether you strongly disagree, disagree, agree, or strongly agree that current US government agencies are adequately prepared for controlling risks from nanotechnologies in the following categories”. This four-point likert scale indexed 1 as ‘strongly disagree’ through 4 as ‘strongly agree’; also provided were the options: “not familiar with relevant agency or its regulations / can’t answer” and “don’t know / not sure”. Figure **1** illustrates the results for each of 14 scenarios, where points on color-coded lines indicate the mean score on agency preparedness for each expert group (NSE, NEHS, and NREG). 

Across 10 of 14 scenarios, the mean scores for all three groups lie to the left of the centerline, demonstrating consistent disagreement with the claim that federal agencies are ‘adequately prepared’ to control risks from nanotechnologies. Agreement is demonstrated in one case: ‘computers and electronic devices’, by just one group (NSE). This result indicates a prevailing trend towards disagreement (*agencies are not prepared for controlling risks*) for a majority of the 14 scenarios presented. The NSE and NEHS groups also visibly vary from scenario to scenario in much closer agreement with one another than with NREG, and differ significantly from one another on only one scenario, again, computers and electronic devices. The NSE and NEHS groups are also proximate to the neutral center for several items (medical devices and treatments, pharmaceuticals, industrial workplaces, chemicals and product additives). More striking, however, are the low mean scores for the NREG group, all of which lie consistently to the left of the NEHS and NSE groups, and largely to the left of the ‘disagree’ point on scale. This suggests that those most fully responsible for managing the risks of nanotechnologies regard government agencies as unprepared, more so than their counterparts outside of government regulatory and research agencies. 

To confirm that the differences in opinion observed between expert groups are statistically significant, we conducted a between-subjects analysis of variance (ANOVA), wherein significant differences in means were found for 12 of 14 scenarios (*p<0.05*). This finding confirms that NREG and NSE groups are most dissimilar in their opinions on a majority of items. Differences in opinion are also observed between the NREG and NEHS groups with fewer significant differences across scenarios, and smaller difference in the magnitude of their mean responses. Detailed results are provided in Tables S2 and S3. 

To determine whether the difference in mean responses across groups is significant when all 14 scenarios are aggregated, we also created a composite measure (hereafter referred to as ‘Preparedness Index’) using Principal Component Analysis (PCA) with orthogonal rotation (Varimax). One component accounting for 56% of the variance was selected using a scree test [36], and index scores were calculated using the Anderson-Rubin method. The resulting Preparedness Index was found to constitute a consistent and highly reliable scale (Chronbach’s alpha = 0.98). We found a statistically significant difference between expert groups using an ANOVA test with the Preparedness Index (F(2, 251) = 10.216, p = < .001), a finding that is consistent with ANOVA results for individual nanotechnology scenarios reported above. A Tukey HSD *post hoc* analysis revealed that the Preparedness Index score was significantly higher for NSE (N=114, 0.21 +/- 0.91; p < .001) and NEHS (N=86, 0.02 +/- 0.85; p < .001) groups than for the NREG (N=54, -0.47 +/- 0.99) group. However, there was no statistically significant difference between NSE and NEHS groups (p = .33). Thus preparedness judgments can be said to differ significantly across NSE and NREG, and NEHS and NREG groups only. 

### Drivers of Perceived Agency Preparedness

Several hypotheses (2 through 5 above) were posed for why experts might differ on their views of agency preparedness. Among those most important to policy discussions is whether or not nanomaterials *really are* seen as different from their bulk form and so necessitate additional capacity and expertise at the level of regulation. Seeing nanotechnologies as ‘new’ or ‘novel’ for the purpose of testing here (hypothesis 2) was operationalized using an index developed for this purpose, measuring seven dimensions of novelty (listed in Table **1** below). The first component labeled “New and Uncertain Risks” (Cronbach’s α = 0.65) is highly correlated with the variables ‘Properties Cannot be Anticipated’, ‘New Risks’, ‘Risks are Not Well Known’, ‘Risks Cannot be Determined’, and ‘More Uncertainty than Bulk Materials’. The second component is associated with ‘New Benefits’ and ‘Novel Properties’, and is labeled accordingly as “Novel Benefits and Properties” (Cronbach’s α = 0.74). The questions designed to elicit these judgments were developed using face-to-face interviews with US and Canadian nanotechnology experts [31], and are summarized in the table. The two orthogonal components were isolated using a PCA with Varimax rotation followed by a scree test, and orthogonal factor scores were calculated using the Anderson-Rubin method. Factor loadings from the PCA are shown in Table **1**. The component “New and Uncertain Risks” is moderately correlated with Agency Preparedness (Pearson’s r = -.479), indicating its potential as a predictor of preparedness perceptions, while “Novel Benefits and Properties” is only slightly correlated (r = -0.01). Both factors are included in the regression analysis below to examine their influence on perceptions of ‘Agency Preparedness’.

**Table 1:** Factor loadings from a principal components analysis over seven ‘novelty’ rating scales, averaged across individuals (varimax rotated solution).

Rating Scale	*Factor* ** *1*: New and Uncertain Risks (*31.9% of var.*)	*Factor* ** *2*: Novel Benefits and Properties (*20.8% of var.*)
New Benefits^1^	.10	**.87**
Novel Properties^2^	.08	**.87**
Properties Cannot be Anticipated^3*^	**.54**	.17
New Risks^4^	**.56**	.24
Risks are Not Well Known^5*^	**.76**	-.16
Risks Cannot be Determined^6*^	**.73**	-.02
More Uncertainty than Bulk Materials^7^	**.56**	.16

*Loadings exceeding 0.3 are in boldface. Items marked with an asterisk are reverse coded to facilitate comparison. For each survey item, the following Likert scale was used: 1 – Strongly Disagree, 2 – Disagree, 3 – Agree, 4 – Strongly Agree.*

Corresponding Survey Questions:

1. Nano-scale materials promise benefits for society that are not possible with bulk (non nano-scale) materials

2. Nano-scale materials possess novel properties that are not expressed in their corresponding bulk forms

3. The novel properties of nano-scale materials cannot be anticipated by knowing the properties of the same material in its bulk form

4. Nano-scale materials pose risks for society that are not present with bulk (non nano-scale) materials

5. The health and environmental risks from nano-scale materials are not well known to scientists

6. The existing methods for assessing health and environmental risks from bulk materials are not suitable for determining risks from nano-scale materials

7. There is more uncertainty about the risks from nano-scale materials than the risks from bulk forms

Next we tested trust (hypothesis 3) as explaining different views of agency preparedness. Trust has been found to be highly predictive of expert and lay views of the risk and regulation of new technologies in general. We developed a comprehensive index based on responses to the question: “Please indicate how trustworthy you feel the following government agencies are for effectively managing nano-specific environmental health and safety risks from: very untrustworthy, somewhat untrustworthy, somewhat trustworthy, very trustworthy”. Federal regulatory agencies presented included those expected to play a central role in nanotechnology regulation[37], including: US Environmental Protection Agency (*EPA*)*, Food and Drug Administration (FDA*)*, Occupational Safety and Health Administration (OSHA*)*, Consumer Product Safety Commission (CPSC*). By evaluating trustworthiness of several regulatory agencies in the aggregate this variable provides a robust measure of trust in regulatory agencies in general. As before, the index was created with all four items using a PCA with orthogonal (varimax) rotation, then calculated using the Anderson-Rubin method. Only one component is needed to explain the correlations between all four items, accounting for 69% of the variance (Cronbach’s α = 0.86). The trust index is moderately correlated with preparedness perceptions (Pearson’s r = .263), and so was also included in the regression.

The degree of responsibility expected from, or attributed to, various stakeholders constituted our fourth predictor of views of preparedness (hypothesis 4). To test this hypothesis we developed an index based on responses to the question: “*For the following list of groups or stakeholders, please indicate whether they should: Bear none of the responsibility, some of the responsibility, most of the responsibility, or all of the responsibility,* *for* *managing* *risks* *that* *emerge* *from* *nanotechnologies*.” Stakeholder groups included:

• Academic basic sciences and R&D laboratories (*ie. Physics, chemistry, engineering*)
• Academic environmental and health sciences laboratories (*ie. toxicology, epidemiology*)
• Private research and development laboratories
• Smaller companies developing nanotechnology products
• Larger companies developing nanotechnology products
• Government agencies (*eg. EPA, FDA*)
• Environmental groups and non-governmental organizations (NGOs)
• Consumers, through their product purchasing decisions

The ‘responsibility’ index provides a measure of whether responsibility is attributed to a single or narrow set of stakeholders (resulting in a lower responsibility score), versus many or all stakeholder groups (higher responsibility score). This ‘responsibility’ index was created as above with all eight items using a PCA with varimax rotation and the Anderson-Rubin method. Only one component is needed to explain the correlations between all eight items, accounting for 42% of the variance (Cronbach’s α = 0.79). The responsibility index is correlated with preparedness perceptions (r = -.182), and hence was included in the regression analysis to test as a predictor variable. 

Finally, we tested the influence of experts’ ‘socio-political values’, measured as *social and economic conservatism*, on their views of agency preparedness (hypothesis 5). Socio-political values were measured using the following two questions: “*The terms* ‘*liberal*’ *and* ‘*conservative*’ *may mean different things to different people, depending on the kind of issue one is considering. In terms of economic issues, would you say you are: 1- Very Liberal, 2 – Somewhat Liberal, 3 – Somewhat Conservative, 4- Very Conservative, 5 - Don’t Know/Not sure*”. The question was then repeated, using *social issues*. A ‘Social/Economic Conservatism’ index was created based on the standardized z-score of the combined mean responses for these two questions (Cronbach’s α= 0.64). The index indicates potential as a predictive variable (Pearson’s r = .127), and so was included in the regression analysis. 

Table **2** provides a summary of descriptive statistics for all four driver variables (novelty, trust, responsibility, and socio-political values) and controls (demographics, domain of expertise) across all three expert groups.

**Table 2:** Descriptive statistics for control and independent variables.

**Variable**	**Category**	**NSE (N=114)**	**NEHS (N=86)**	**NREG (N=54)**
***Demographic Variables***				
Graduation Year (*mean (SD*))		1990 (11.7)	1994 (10.8)	1992 (8.9)
Gender (*% male*)		89.30%	60.00%	68.50%
Education	*PhD*	99.10%	98.80%	50.90%
	*Masters*	0.90%	0.00%	30.90%
	*Bachelors*	0.00%	1.20%	18.20%
***Domain of Expertise Variables***				
Discipline	*Physical Sciences*	92.10%	30.20%	16.40%
	*Other*	7.90%	69.80%	83.60%
Affiliation	*Academic*	82.30%	90.60%	0.00%
	*Government*	*7.10%*	*0.00%*	*96.40%*
	*Other*	10.60%	9.40%	3.60%
***Index Variables***				
Trust (*mean (SD*))		*-0.08 (0.95)*	*-0.06 (0.92)*	*-0.08 (1.16)*
Responsibility (*mean (SD*))		-0.1 (0.93)	-0.02 (0.95)	0.25 (1.33)
Conservatism (*mean (SD*))		1.42 (0.39)	1.47 (0.37)	1.52 (0.43)
Novelty-Risks (*mean (SD*))		0.33 (0.91)	-0.19 (0.95)	-0.4 (1.0)
Novelty-Benefits (*mean (SD*))		0.08 (0.97)	-0.09 (0.97)	-0.01 (1.1)

All values for Demographics and Domain of Expertise variables indicate the distribution of respondents by expert group (out of a total of 100%), while figures for ‘Graduation Year’ specify means and standard deviations. Values for independent variables trust, responsibility, conservatism, and novelty indicate mean index scores and standard deviations.

To test the influence of the above four constructed independent variables on perceptions of regulatory agency preparedness for managing risks, a hierarchical ordinary least squares (OLS) multivariate regression was conducted. Using our composite ‘Preparedness Index’ as the dependent variable, independent variables were entered in six steps to investigate their relative power in predicting agency preparedness. Step 1 introduces ‘expert group’ along with demographic variables gender, education, and year of highest degree (as a proxy for age) as control variables. Step 2 introduces ‘domain of expertise’ control variables, including *disciplinary field*, and *Institutional Affiliation*. Steps 3 through 6 introduce the socio-political values, trust, responsibility, and novelty variables respectively. Table **3** presents the results of the hierarchical regression.

**Table 3:** Hierarchical regression analysis with preparedness index as dependent variable.

**Variable**	**B**	**S.E.**	**β**
(Constant)	0.25	0.24	
*Step 1. Demographics and Group*			
NSE vs. NEHS^a^	0.09	0.15	0.05
NSE vs. NREG	-0.27	0.3	-0.12
Gender^b^	0.03	0.12	0.01
Education^c^	-0.31	0.21	-0.1
Year of Degree^d^	0.02	0.06	0.02
*Step 2. Domain of Expertise*			
Disciplinary Field^e^	-0.13	0.27	-0.06
Affiliation (*Academic vs Government*)^f^	0.17	0.18	0.05
Affiliation (*Academic vs Other*)	0.18	0.14	0.1
*Step 3. Socio-Political Values*			
Social/Economic Conservatism^g^	0.16**	0.06	0.15**
*Step 4. Trust*			
Trust^h^	0.20***	0.05	0.21***
*Step 5. Responsibility*			
Responsibility^i^	-0.13**	0.05	-0.14**
*Step 6. Nanotechnology Novelty*			
Novelty: New and Uncertain Risks^j^	-0.40***	0.06	-0.40***
Novelty: Novel Benefits and Properties^k^	-0.03	0.05	-0.04

N=254. *p <.05. **p <.01. ***p <.001. R^2^ = .06 for Step 1; *Δ*R^2^ = .02 for Step 2 (p = .11); *Δ*R^2^ = .02 for Step 3 (p = 0.02); *Δ*R^2^ = .06 for Step 4 (p < .001); *Δ*R^2^ = .03 for Step 5 (p < .01); *Δ*R^2^ = .14 for Step 6 (p < .001) . Total adjusted R^2^ = 0.32

Cell entries for Steps 1 through 6 are final unstandardized (B) and standardized (β) regression coefficients. Diagnostics indicate no evidence of multicollinearity (VIF < 10), and that none of the four principal assumptions for linear regressions have been violated [36].

a Paired dummy variables, where ‘NSE vs NEHS’ is coded as NSE = 0, NEHS =1, and ‘NSE vs NREG’ is coded as NSE = 0, NREG = 1.

b 1 = female, 0 = male

c 1 = PhD, 0 = Bachelors/Masters

d *Standardized continuous variable*

e 1 = physical sciences, 0 = other, where ‘physical sciences’ includes chemistry, physics, materials science, chemical engineering, electrical engineering, and mechanical engineering

f Paired dummy variables, where ‘academic vs government’ is coded as academic = 0, government = 1, and ‘academic vs other’ is coded as academic = 0, other = 1.

g ^*h*^ *,* ^*i*^ *,* ^*j*^ *,* ^*k*^ *Continuous index variables, described above*

The resulting final model explained 32% of the variance (*adjusted R* ^*2*^) and revealed significant contributions (*at the p < .05 level*) from four variables: “Novelty: New and Uncertain Risks” (β = -0.40*; p < .001, ΔR* ^2^ = 14%), “Trust” (β = 0.21*; p < .001, ΔR* ^2^ = 6%), “Responsibility” (β = -0.14*; p < .01, ΔR* ^2^ = 3%), and “Social/Economic Conservatism” (β = 0.15*; p = .02, ΔR* ^2^ = 2%). We see that respondents judged agencies are *more* prepared when they were more conservative, and when they had more trust in regulatory agencies. Conversely, respondents judged agencies are *less* prepared when they attributed responsibility more uniformly across stakeholder groups, and when they perceived nanotechnology risks as new and more uncertain. Experts relied strongly upon framing of risks (as novel) as a heuristic cue, a finding that empirically demonstrates the link between novelty and risk perceptions, and expands upon recent interview-based research showing substantial differences in experts’ framing of the novelty of nanotechnology risks [32]. The framing of benefits and properties as novel however was not utilized as a heuristic cue, and little variation in views of the novelty of benefits were found between groups (see Table **2**). Thus hypothesis 2, that perceptions of novelty significantly affect preparedness perceptions, is supported for the novelty of nanomaterial *risks*, but not for novelty of properties and benefits. 

Trust in regulatory agencies was also a strong driver of preparedness perceptions, supporting hypothesis 3, and reinforcing findings in the risk literature that demonstrate a significant inverse relationship between trust and perceived risk [25]. However, no significant difference in means was observed across groups for this variable (see Table **2**). This suggests that trust is limited to ‘within-group’ variation and does not drive observed differences in preparedness perceptions between groups. ‘Trust in regulatory agencies’ can be understood to reflect several possible trust judgments, so it is important to assess what aspect of trust is being invoked. Trust in regulatory agencies to manage risks may reflect, among others, 1) trust in regulatory agencies’ *intent* to manage risks, 2) trust that regulatory authority and *regulatory mechanisms are adequate* for the task, or 3) trust that regulators have *adequate evidence* and a sound scientific basis to take action. A strong inverse correlation between trust and novelty of risks would provide evidence for cases 2 and 3, where the novelty of nanotechnology risks challenge the adequacy of evidence, or appropriateness of existing regulatory mechanisms and authority. We found a small but significant negative correlation between the aggregate metrics of trust and novelty of risk (Pearson’s r = -.128, p < .05, 2-tailed), suggesting that our trust metric is based in part on judgments of regulatory adequacy for managing nanomaterial risks. 

Attribution of collective stakeholder responsibility was also found to relate significantly to views on preparedness, supporting hypothesis 4. The attributed responsibility index provides insight into expert’s expectations for stakeholders to manage risks. A high score on the attributed responsibility scale indicates that a greater degree of responsibility is expected from stakeholders overall. It also reflects the judgment that a wide range of stakeholders should play a role in the management of nanotechnologies, rather than one or a narrow set of stakeholders. Attributed responsibility can thus be seen as a proxy measurement for perceived *magnitude* or *complexity* of the risk management challenge, where a greater challenge requires greater attention from a number of stakeholders. Hence, when attributed responsibility is high, the management challenge is seen as great, and regulatory agencies (among other stakeholder groups) are perceived as less prepared for managing those risks on their own. Nonetheless, attributed responsibility played only a minor role in overall variance explained by the model (*ΔR* ^2^ = 3%). 

The finding on the significant role of social-political views (conservatism) is somewhat contrary to theory suggesting that experts draw upon their expertise and experience, and not upon heuristic cues and value predispositions, when making judgments on risk and regulatory policy [23,24]. The range of socio-political differences across the three groups is small with mean responses roughly half-way between ‘very liberal’ and ‘liberal’. Nonetheless, the regression results weakly support hypothesis 5, and reflect longstanding findings that cultural worldviews, including political ideology, influence expert judgment [10]. Our results also echo recent findings in the nano-risk perceptions literature, where Corley et al. found economic conservatism is inversely related to experts’ support for regulation of nanotechnology [22].

## Discussion

Consistent differences exist between expert groups in their views on agency preparedness to manage nanotechnology risks, yet all three groups perceive regulatory agencies as unprepared. What is most striking however is that NREG experts see regulatory agencies as considerably less prepared than do their NSE or NEHS counterparts. Taking a closer look, the drivers of experts’ concerns over regulator preparedness tell a more nuanced story. After accounting for other differences, the ‘expert group’ classification *per se* does not drive the observed differences in preparedness perceptions. Rather a substantial portion of this difference results from differing assessments of the perceived novelty of risks across expert groups. Of the remaining variables, trust in regulators is a small but significant driver, and our findings suggest a link between concerns over the novelty of nanomaterials and the adequacy of regulatory design. Experts’ views on stakeholder responsibility are not particularly surprising since greater reliance on a collective responsibility model would need the burden to move away exclusively from regulatory bodies to other groups, and result presumptively in a reduced sense of preparedness. 

Experts’ reliance in part upon socio-political values indicates that personal values also play a minor role in preparedness judgments. This might indicate some difficulty with the evaluation task, where a greater reliance upon personal values can be expected for experts who make judgments that span beyond their specific area of expertise [10,20-22]. For instance, experts outside of regulatory agencies may have less direct knowledge and experience with the challenges of regulation and hence may rely in part upon personal values and experiences when making an assessment (and vice versa) thus accounting for some of the observed variance in preparedness judgments. 

While these four factors (novelty of risks, socio-economic views, trust, and attributed responsibility) provide insight into the drivers of preparedness perceptions, together they account for approximately one-third of the observed variance. The differences in mean preparedness judgments between NREG, NSE, and NEHS groups (in Figure 1) can likely be explained by a combination of the above factors, optimism bias owing to an experts’ proximity to the development of new technologies, and other unmeasured factors including an experts’ depth of understanding of the limitations of risk assessment methodologies and regulatory challenges in general. For instance, experts in regulatory agencies may be more keenly aware of historical successes and failures in managing risks under uncertainty, as well as the new challenges inherent in regulating emerging (and highly uncertain) nanotechnologies, than other expert groups. Recent interviews conducted with experts in US Federal regulatory agencies [38] indeed point to limited scientific knowledge and uncertainty surrounding nanomaterial behavior as perceived complicating factors for risk assessors and regulators. Given their close familiarity with matters of regulation, NREG participants may be better suited to judge regulatory agency preparedness. Conversely, their close proximity to regulatory matters may also result in its own bias, whereby NREG experts may focus too narrowly on risk and ‘miss the forest for the trees’. These findings point to a need for decision makers to solicit opinions from a wide range of experts along the nanomaterial life cycle, from upstream research to downstream management, in matters of risk regulation.

## Supporting Information

Table S1 **Agencies involved in NREG sample selection.**
(DOCX)

Table S2 **One-Way Analysis of Variance (ANOVA) measuring the significance of differences in** **‘agency preparedness’ ratings by expert group for 14 nanotechnology scenarios (scale: ‘1- strongly disagree’, ‘2 – disagree’, ‘3 – agree’, ‘4 – strongly agree’)**. ANOVA results are presented where the assumption of homogeneity of variances of groups was maintained. Welch test results are presented in place of ANOVA results for variables with non-homogeneous variances.
(DOCX)

Table S3 **Games-Howell post hoc analysis indicating significant differences in means between NSE-NEHS, NSE-NREG, and NEHS-NREG group pairings.**
(DOCX)
