# Supplementary material for: Expert Views on Regulatory Preparedness for Managing the Risks of Nanotechnologies
Source: PLoS One. 2013 Nov 11;8(11):e80250. doi: 10.1371/journal.pone.0080250 (PMC3823619; doi:10.1371/journal.pone.0080250)
Supplement: Table S1 — Agencies involved in NREG sample selection. (DOCX) [file pone.0080250.s001.docx]

**TABLE S1.** *Agencies involved in NREG sample selection*

| ***Federal level regulatory agencies*** |
| --- |
| EPA – Environmental Protection Agency – National Offices, Region 1, Region 5 |
| FDA – US Food and Drug Administration |
| OSHA – US Occupational Safety and Health Administration |
| USDA – US Department of Agriculture |
| ***Other federal level (non-regulatory) agencies, labs, and institutes involved in nano risk research and regulation*** |
| NIH – National Institutes of Health |
| NIOSH – National Institute for Occupational Safety and Health |
| NIST – National Institutes of Standards and Technology |
| ANL - Argonne National Lab |
| BNL - Brookhaven National Lab |
| LANL – Los Alamos National Lab |
| LBL – Lawrence Berkeley Lab |
| LLNL – Lawrence Livermore National Lab |
| NCI - National Cancer Institute |
| ORNL - Oak Ridge National Lab |
| PNNL – Pacific Northwest National Lab |
| Air Force, Army, Navy |
| Ames Laboratory |
| NSF - National Science Foundation |
| ***State level agencies*** |
| California EPA – DTSC – Department of Toxic Substances Control |
| Massachusetts Department of Environmental Protection |
| Massachusetts Department of Labor |
| Massachusetts Department of Public Health |
| Massachusetts Division of Occupational Safety |
| Massachusetts Office of Business Development |
| Massachusetts Office of Technical Assistance and Technology |
| North Carolina NCDENR - NC Dept. of Environment and Natural Resources |
| New York Department of Environmental Conservation |
| New York Department of Health |
| Washington Department of Ecology |
