# Supplementary material for: Expert Views on Regulatory Preparedness for Managing the Risks of Nanotechnologies
Source: PLoS One. 2013 Nov 11;8(11):e80250. doi: 10.1371/journal.pone.0080250 (PMC3823619; doi:10.1371/journal.pone.0080250)
Supplement: Table S2 — One-Way Analysis of Variance (ANOVA) measuring the significance of differences in ‘agency preparedness’ ratings by expert group for 14 nanotechnology scenarios (scale: ‘1- strongly disagree’, ‘2 – disagree’, ‘3 – agree’, ‘4 – strongly agree’). ANOVA results are presented where the assumption of homogeneity of variances of groups was maintained. Welch test results are presented in place of ANOVA results for variables with non-homogeneous variances. (DOCX) [file pone.0080250.s002.docx]

**TABLE S2.**  *One-Way Analysis of Variance (ANOVA) measuring the significance of differences in ‘agency preparedness’ ratings by expert group for 14 nanotechnology scenarios (scale: ‘1- strongly disagree’, ‘2 – disagree’, ‘3 – agree’, ‘4 – strongly agree’). ANOVA results are presented where the assumption of homogeneity of variances of groups was maintained. Welch test results are presented in place of ANOVA results for variables with non-homogeneous variances.*

| **Nanotechnology Scenario** | **GROUP** | **N** | **Mean** | **S.D.** | **Levene Test^a^** | | **ANOVA** |  |
| --- | --- | --- | --- | --- | --- | --- | --- | --- |
|  |  |  |  |  | **Levene Statistic** | **p-value** | **F-value** | **p-value** |
| Industrial workplaces | NSE | 78 | 2.55 | 0.907 | 2.553 | 0.08 | 10.067 | 0 |
|  | NEHS | 78 | 2.47 | 0.785 |  |  |  |  |
|  | NREG | 53 | 1.92 | 0.781 |  |  |  |  |
|  | Total | 209 | 2.36 | 0.867 |  |  |  |  |
| Pharmaceuticals | NSE | 83 | 2.55 | 0.845 | 0.572 | 0.565 | 4.974 | 0.008 |
|  | NEHS | 79 | 2.49 | 0.766 |  |  |  |  |
|  | NREG | 44 | 2.09 | 0.858 |  |  |  |  |
|  | Total | 206 | 2.43 | 0.834 |  |  |  |  |
| Medical devices and treatments | NSE | 82 | 2.61 | 0.766 | 0.026 | 0.975 | 3.481 | 0.033 |
|  | NEHS | 77 | 2.53 | 0.718 |  |  |  |  |
|  | NREG | 42 | 2.24 | 0.79 |  |  |  |  |
|  | Total | 201 | 2.5 | 0.762 |  |  |  |  |
| Industrial releases to the environment (air, water, soil) | NSE | 82 | 2.21 | 0.766 | 1.295 | 0.276 | 3.738 | 0.025 |
|  | NEHS | 76 | 2.07 | 0.718 |  |  |  |  |
|  | NREG | 52 | 1.85 | 0.751 |  |  |  |  |
|  | Total | 210 | 2.07 | 0.755 |  |  |  |  |
| Food and food packaging | NSE | 81 | 2.36 | 0.795 | 0.861 | 0.425 | 5.822 | 0.004 |
|  | NEHS | 72 | 2.21 | 0.749 |  |  |  |  |
|  | NREG | 43 | 1.86 | 0.774 |  |  |  |  |
|  | Total | 196 | 2.19 | 0.793 |  |  |  |  |
| Environmental releases (air, water, soil) from consumer products | NSE | 80 | 2.14 | 0.742 | 2.124 | 0.122 | 4.988 | 0.008 |
|  | NEHS | 77 | 1.95 | 0.667 |  |  |  |  |
|  | NREG | 53 | 1.74 | 0.763 |  |  |  |  |
|  | Total | 210 | 1.97 | 0.735 |  |  |  |  |
| Computers and electronic devices | NSE | 83 | 2.87 | 0.712 | 1.158 | 0.316 | 16.863 | 0 |
|  | NEHS | 70 | 2.36 | 0.66 |  |  |  |  |
|  | NREG | 47 | 2.19 | 0.77 |  |  |  |  |
|  | Total | 200 | 2.53 | 0.763 |  |  |  |  |
| Vitamins and supplements | NSE | 72 | 2.1 | 0.754 | 0.762 | 0.468 | 7.547 | 0.001 |
|  | NEHS | 74 | 1.92 | 0.636 |  |  |  |  |
|  | NREG | 42 | 1.6 | 0.544 |  |  |  |  |
|  | Total | 188 | 1.91 | 0.688 |  |  |  |  |
| Environmental remediation (contaminated site cleanup) | NSE | 77 | 2.42 | 0.732 | 0.556 | 0.574 | 2.598 | 0.077 |
|  | NEHS | 72 | 2.32 | 0.668 |  |  |  |  |
|  | NREG | 46 | 2.11 | 0.795 |  |  |  |  |
|  | Total | 195 | 2.31 | 0.731 |  |  |  |  |
| Waste products and contaminated sites | NSE | 77 | 2.38 | 0.744 | 2.471 | 0.087 | 7.766 | 0.001 |
|  | NEHS | 75 | 2.31 | 0.657 |  |  |  |  |
|  | NREG | 48 | 1.9 | 0.66 |  |  |  |  |
|  | Total | 200 | 2.24 | 0.716 |  |  |  |  |
| **Nanotechnology Scenario** | **GROUP** | **N** | **Mean** | **S.D.** | **Levene Test^a^** | | **Welch Test^c^** | |
|  |  |  |  |  | **Levene Statistic** | **Sig.** | **Statistic^b^** | **Sig.** |
| Cosmetics | NSE | 68 | 2.18 | 0.828 | 4.475 | 0.013 | 2.855 | 0.062 |
|  | NEHS | 72 | 1.97 | 0.671 |  |  |  |  |
|  | NREG | 48 | 1.83 | 0.724 |  |  |  |  |
|  | Total | 188 | 2.01 | 0.753 |  |  |  |  |
| Pesticides and agricultural applications | NSE | 75 | 2.28 | 0.894 | 3.704 | 0.026 | 3.738 | 0.027 |
|  | NEHS | 72 | 2.39 | 0.761 |  |  |  |  |
|  | NREG | 48 | 2 | 0.772 |  |  |  |  |
|  | Total | 195 | 2.25 | 0.827 |  |  |  |  |
| Chemicals and product additives | NSE | 80 | 2.48 | 0.795 | 3.11 | 0.047 | 11.066 | 0 |
|  | NEHS | 74 | 2.23 | 0.653 |  |  |  |  |
|  | NREG | 49 | 1.82 | 0.755 |  |  |  |  |
|  | Total | 203 | 2.23 | 0.776 |  |  |  |  |
| Other consumer products | NSE | 56 | 2.29 | 0.756 | 3.657 | 0.028 | 3.426 | 0.036 |
|  | NEHS | 65 | 2 | 0.637 |  |  |  |  |
|  | NREG | 47 | 1.94 | 0.734 |  |  |  |  |
|  | Total | 168 | 2.08 | 0.718 |  |  |  |  |
| a. Levene Test for Homogeneity of Variances | | | |  |  |  |  |  |
| b. Asymptotically F distributed. | |  |  |  |  |  |  |  |
| c. Welch Test for Equality of Means | | |  |  |  |  |  |  |
