# Supplementary material for: Expert Views on Regulatory Preparedness for Managing the Risks of Nanotechnologies
Source: PLoS One. 2013 Nov 11;8(11):e80250. doi: 10.1371/journal.pone.0080250 (PMC3823619; doi:10.1371/journal.pone.0080250)
Supplement: Table S3 — Games-Howell post hoc analysis indicating significant differences in means between NSE-NEHS, NSE-NREG, and NEHS-NREG group pairings. (DOCX) [file pone.0080250.s003.docx]

**TABLE S3.** *Games-Howell post hoc analysis indicating significant differences in means between NSE-NEHS, NSE-NREG, and NEHS-NREG group pairings.*

| **Dependent Variable** | **(I) GROUP** | **(J) GROUP** | **Mean Difference (I-J)** | **Std. Error** | **p-value** | **95% Confidence Interval** |  |
| --- | --- | --- | --- | --- | --- | --- | --- |
|  |  |  |  |  |  | **Lower Bound** | **Upper Bound** |
| Cosmetics | NSE | NEHS | 0.204 | 0.128 | 0.25 | -0.1 | 0.51 |
|  |  | NREG | 0.343 | 0.145 | 0.05 | 0 | 0.69 |
|  | NEHS | NREG | 0.139 | 0.131 | 0.54 | -0.17 | 0.45 |
| Pesticides and agricultural applications | NSE | NEHS | -0.109 | 0.137 | 0.71 | -0.43 | 0.21 |
|  |  | NREG | 0.28 | 0.152 | 0.16 | -0.08 | 0.64 |
|  | NEHS | NREG | .389* | 0.143 | 0.02 | 0.05 | 0.73 |
| Industrial workplaces | NSE | NEHS | 0.077 | 0.136 | 0.84 | -0.24 | 0.4 |
|  |  | NREG | .627* | 0.148 | 0 | 0.27 | 0.98 |
|  | NEHS | NREG | .550* | 0.139 | 0 | 0.22 | 0.88 |
| Pharmaceuticals | NSE | NEHS | 0.061 | 0.127 | 0.88 | -0.24 | 0.36 |
|  |  | NREG | .463* | 0.159 | 0.01 | 0.08 | 0.84 |
|  | NEHS | NREG | .403* | 0.155 | 0.03 | 0.03 | 0.77 |
| Medical devices and treatments | NSE | NEHS | 0.077 | 0.118 | 0.79 | -0.2 | 0.36 |
|  |  | NREG | .372* | 0.148 | 0.04 | 0.02 | 0.73 |
|  | NEHS | NREG | 0.294 | 0.147 | 0.12 | -0.06 | 0.65 |
| Industrial releases to the environment (air, water, soil) | NSE | NEHS | 0.142 | 0.118 | 0.46 | -0.14 | 0.42 |
|  |  | NREG | .361* | 0.134 | 0.02 | 0.04 | 0.68 |
|  | NEHS | NREG | 0.22 | 0.133 | 0.23 | -0.1 | 0.54 |
| Food and food packaging | NSE | NEHS | 0.15 | 0.125 | 0.46 | -0.15 | 0.45 |
|  |  | NREG | .498* | 0.147 | 0 | 0.15 | 0.85 |
|  | NEHS | NREG | 0.348 | 0.147 | 0.05 | 0 | 0.7 |
| Environmental releases (air, water, soil) from consumer products | NSE | NEHS | 0.189 | 0.112 | 0.22 | -0.08 | 0.46 |
|  |  | NREG | .402* | 0.134 | 0.01 | 0.08 | 0.72 |
|  | NEHS | NREG | 0.212 | 0.129 | 0.23 | -0.1 | 0.52 |
| Computers and electronic devices | NSE | NEHS | .510* | 0.111 | 0 | 0.25 | 0.77 |
|  |  | NREG | .676* | 0.137 | 0 | 0.35 | 1 |
|  | NEHS | NREG | 0.166 | 0.137 | 0.45 | -0.16 | 0.49 |
| Vitamins and supplements | NSE | NEHS | 0.178 | 0.116 | 0.27 | -0.1 | 0.45 |
|  |  | NREG | .502* | 0.122 | 0 | 0.21 | 0.79 |
|  | NEHS | NREG | .324* | 0.112 | 0.01 | 0.06 | 0.59 |
| Environmental remediation (contaminated site cleanup) | NSE | NEHS | 0.096 | 0.115 | 0.68 | -0.18 | 0.37 |
|  |  | NREG | 0.307 | 0.144 | 0.09 | -0.04 | 0.65 |
|  | NEHS | NREG | 0.211 | 0.141 | 0.3 | -0.13 | 0.55 |
| Waste products and contaminated sites | NSE | NEHS | 0.07 | 0.114 | 0.81 | -0.2 | 0.34 |
|  |  | NREG | .481* | 0.128 | 0 | 0.18 | 0.78 |
|  | NEHS | NREG | .411* | 0.122 | 0 | 0.12 | 0.7 |
| Chemicals and product additives | NSE | NEHS | 0.245 | 0.117 | 0.09 | -0.03 | 0.52 |
|  |  | NREG | .659* | 0.14 | 0 | 0.33 | 0.99 |
|  | NEHS | NREG | .413* | 0.132 | 0.01 | 0.1 | 0.73 |
| Other consumer products | NSE | NEHS | 0.286 | 0.128 | 0.07 | -0.02 | 0.59 |
|  |  | NREG | 0.35 | 0.147 | 0.05 | 0 | 0.7 |
|  | NEHS | NREG | 0.064 | 0.133 | 0.88 | -0.25 | 0.38 |
| *. The mean difference is significant at the 0.05 level. | | | |  |  |  |  |
